# Gender differences in health-related quality of life, blood pressure and heart rate among patients with paroxysmal atrial fibrillation after performing MediYoga

**DOI:** 10.1016/j.ijcha.2023.101274

**Published:** 2023-09-22

**Authors:** Maria Wahlström, Jörgen Medin, Monica Rydell Karlsson

**Affiliations:** a Karolinska Institutet, Department of Clinical Sciences, Department of Cardiology, Danderyd Hospital, Stockholm, Sweden; b Department of Health Promoting Science, Sophiahemmet University, Box 5035, 114 86 Stockholm, Sweden; c Ersta Sköndal Bräcke University College, Stockholm, Sweden; d Department of Health Sciences, Mid Sweden University, Östersund, Sweden

**Keywords:** Gender, Paroxysmal atrial fibrillation, Quality of life, Blood pressure, Heart rate, Yoga

## Abstract

**Introduction:**

Patients with paroxysmal atrial fibrillation experience low health-related quality of life which can be improved by performing yoga. The aim of this study was to evaluate gender differences in health-related quality of life, blood pressure and heart rate among patients with paroxysmal atrial fibrillation after performing MediYoga.

**Methods:**

This is a secondary analysis of subgroups, investigating the yoga groups, from two randomized controlled trials (RCT 1: yoga group versus control group, RCT 2: a three-armed randomized study with yoga, control and relaxation groups). The yoga groups performed MediYoga for one hour/week over a 12-week period in both studies. Quality of life (SF-36), blood pressure and heart rate were collected at baseline and end of study.

**Results:**

No differences were found between the women and men. Within the women’s group, there were improvements in vitality (*p* = 0.011), social function (*p* = 0.022), mental health (*p* = 0.007) and Mental Components Summary (*p* = 0.022). There were differences within the men’s group in bodily pain (*p* = 0.005), general health (*p* = 0.003), vitality (*p* = 0.026), social function (*p* = 0.005), role-emotion (*p* = 0.011) and Mental Components Summary (*p* = 0.018). Within the women’s group, differences were observed in systolic blood pressure (*p* = 0.010) and diastolic blood pressure (*p* = <0.001). The men’s group also showed improvement in diastolic blood pressure (*p* = 0.021).

**Conclusion:**

MediYoga improved mental health as well as diastolic blood pressure in both men and women with PAF. This study suggests that both men and women, with PAF, may benefit from complementary treatment such as yoga.

**Clinical Trial Gov Id:** NCT01789372.

## 1 Introduction

Atrial fibrillation (AF) is the most common heart arrythmia in the adult population and is increasing globally. Modifiable risk factors associated with AF are hypertension, diabetes, hyperlipidaemia, smoking, obesity and high alcohol intake [1]. Paroxysmal atrial fibrillation (PAF) is one of three subgroups of AF and is associated with decreased health-related quality of life (HRQoL) [2]. Patients with PAF report symptoms, such as palpitations and emotions, which have an impact on working habits and social life [3] as well as physical and mental health [4]. Furthermore, gender differences among patients with atrial fibrillation (AF) have been described. Women seem to experience more symptoms, such as palpitations and fear/anxiety [5], [6]. Overall scores of health status, psychological and mental domains in HRQoL are lower in women [5].

The standard treatment for PAF, rate/rhythm regulation and ablation, is not always sufficient and may be complicated, and patients seek complementary methods/treatments [7]. Nowadays, yoga is a well-known complementary method and has been suggested to improve the balance of the parasympathetic and sympathetic nervous systems [8]. Yoga is reported to improve HRQoL, in patients with PAF [9] and decrease symptoms of depression as well as episodes of AF. It is suggested as a complementary method to manage symptoms during AF [10]. In relation to other heart diseases, yoga has been shown to increase HRQoL in patients with hypertension and heart failure [11], [12].

There is a long tradition for patients, both men and women, with cardiovascular diseases to be included in a secondary prevention (which include physical therapy, life-style modifications and long-term follow up) [13]. Recently European Society of Cardiology guidelines (ESC) underline the importance of establishing secondary prevention for patients with AF [1]. The program shall include a multidisciplinary team in health care as a complement to treatment, to help the patients to reduce modifiable risk factors as well as increase HRQoL [1]. The literature suggests that yoga seems to have potential to be included in strategies for secondary prevention as a life-style therapy for patients with AF [14], [15]. With the aim to implement yoga as a complementary treatment for patients with PAF, in health care, it is important to evaluate if both genders will have benefit.

The aim of this study was to assess gender and within gender differences in patients with PAF undergoing MediYoga treatment.

## 2 Material and methods

This is a secondary analysis of subgroups, investigating the yoga groups, from two randomized controlled trials [16] conducted 2009–2012 [9], and 2014–2016 [17], respectively, at Danderyd University Hospital, Stockholm, Sweden.

In the original studies, the patients were screened through medical records, at the arrhythmia department’s out-patient clinic (in the hospital) or referrals from one other arrhythmia department. The first study consisted of 80 patients randomized into a yoga or control group [9]. The second study was three- armed, stratified in gender, consisting of 132 patients randomized to yoga, relaxation or control groups [17]. The inclusion criteria in both studies were a diagnosis of PAF, either early or new diagnosis, and pharmacological treatment for at least three months. Additional inclusion criteria in the second study were symptomatic PAF, with at least one episode of symptomatic AF during the past six months, which had been verified by electrocardiogram (ECG).

In both studies, patients with multiple concurrent serious medical conditions (i.e., advanced cancer, heart failure and renal failure with symptoms), cognitive dysfunction who were considered to have too many difficulties to perform yoga in a group session, and patients with difficulties understanding the Swedish language were excluded. Patients with a diabetes mellitus diagnosis and with an untreated hyperthyroidism were excluded from the second study.

A review of medical records of the participants in the first study was conducted and those without symptomatic AF at the time of inclusion were excluded in this secondary analysis.

**Fig. 1:**
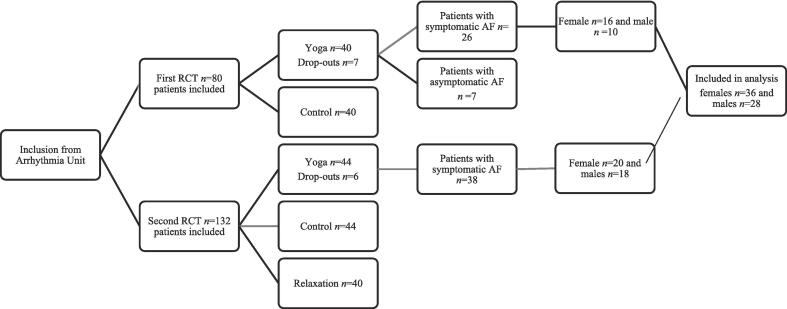
Inclusion and flow-chart.

All patients having undergone yoga treatment formed a dataset for the current study and were divided into two groups (according to gender). A flow chart is presented in Fig. 1.

In both studies, information was provided by letter from the principal author (PA) to those who fulfilled the inclusion criteria. The patients received a telephone call from the PA, after one to two weeks, asking if they wanted to participate in the studies. The participants attended two visits (to the PA), at baseline and after 12 weeks (visit window + 2 weeks), after completing the intervention. Data (i.e., demographics, HRQoL assessments [SF-36], systolic blood pressure [SBP], diastolic blood pressure [DBP] and heart rate [HR]) were collected at both visits by the PA and were evaluated in the same way in the studies. Information regarding medication was obtained from the medical records and confirmed by the participants at the end of studies. The same venue was used for all visits and there were no changes over time.

### 2.1 Intervention

All patients (in both studies) participated in MediYoga (MY, https://mediyoga.se/) which is a therapeutic yoga form. The latter has evolved from Kundalini Yoga which is based on breathing technique, movements, and meditation. The yoga program, used in those studies, comprises of five movements together with deep breathing and meditation, which is described in Appendix 1. MediYoga was performed in group sessions, for one hour, once a week for 12 weeks at the hospital (standard treatment consisting of rhythm/rate regulation and/or ablation was also given). All sessions were conducted by a qualified MY therapist, who had been leading yoga programs for 20 years and MY programs for 6 years. The MY group received a CD-record and a written manual of the yoga program and were encouraged, but not obliged, to perform MY at home. The participants recorded the amount of home practice in a diary, which they were given at the first visit and left with the PA at the second visit.

### 2.2 Comparing groups

The interventions for those groups are not included in the analysis of this study and are reported elsewhere [9], [17].

### 2.3 Assessments

HRQoL was measured by the Short Form Health Survey (SF-36) which contains 36 questions [18], [19] divided into eight subscales; physical functioning (PF), role-physical (RP), bodily pain (BP), general health (GH), vitality (VT), social functioning (SF), role-emotional (RE), mental health (MH) and two domains; Physical Components Summary (PCS) and Mental Components Summary (MCS), which are coded into scores 0–100 where 100 represents the best state of HRQoL. SF-36 is a generic questionnaire and has been found reliable and valid for patients with atrial fibrillation [20].

Blood pressure and HR were measured, after the participant had a resting period of five minutes, with an automatic meter, Omron HEM-711DLXCAN.

### 2.4 Ethics

All participants provided verbal and written informed consent at baseline. The study was approved by the Regional Ethics Committee of Stockholm, Sweden (DNR 2008/1983-31/2, 2013/953-31/4) and is registered in Clinical Trial Gov Id: NCT01789372 and NCT02223156. The investigation conforms to the principles outlined in the Declaration of Helsinki (https://www.wma.net).

### 2.5 Statistical analysis

To strengthen the validity of the analysis, between and within the groups, a linear regression analysis was performed within PCS (SF-36), MCS (SF-36) and gender, in the two studies, and there was no interaction between the two groups (gender) [16].

Categorical variables are presented in number and percent, and continuous variables are presented in mean and standard deviation (SD). Comparisons between the two groups (men and women) were performed with chi-square tests for proportions and one-way ANOVA for analysis of SF-36 (subscales and domains), blood pressure and HR. The Student’s *t*-test was used for within-groups analyses. A significance level of *p* < 0.05 was chosen, between and within groups. The variables in this study have been processed statistically using the data analysis program, IBM SPSS statistics version 27 (IBM SPSS Statistics, IBM Corporation, Armonk, New York).

## 3 Results

The participants in the current study totalled 64 patients with symptomatic AF who completed MediYoga as an active intervention, 36 (56 %) women and 28 (44 %) men. The mean age for women was 66 ± 8 years and 62 ± 9 years for men.

More women, 22 (61 %), had higher hypertension than men, 12 (43 %) but this difference was not statistically significant. There was also no statistical significance in the MY attendance rate; women’s median 10 (5–12) times and men’s median 9.5 (7–12) times.

None of the participants had the need to go to the hospital for chemical reversal and/or cardio-version, of PAF, prior to the inclusion or during in the studies and none received ablation during the studies. Also, none of the participants in the studies reported previous experiences of performing yoga and there were no adverse events reported by the groups. Clinical characteristics are shown in Table 1.

**Table 1:** Clinical characteristics and medications.

	Female *n* = 36	Men *n* = 28	Between groups *p*-value
Age	66 ± 8	62 ± 9	0.343
Hypertension	22 (61)	12 (43)	0.115
Heart failure	0 (0)	0 (0)	–
MI	0 (0)	0 (0)	–
Stroke/TIA	1 (3)	1 (4)	0.688
Beta blockers	26 (72)	17 (61)	0.240
Calcium antagonist	9 (25)	8 (29)	0.484
Antiarrhythmics	13 (36)	10 (36)	0.592
Warfarin	16 (44)	10 (36)	0.328
DOAC	1 (3)	8 (29)	0.335
ASA (aspirin)	5 (14)	4 (14)	0.597
Digoxin	1 (3)	1 (4)	0.688
ACE inhibitor	12 (33)	10 (36)	0.525

Values are mean and standard deviation, n ( %). MI; myocardial infarction. ACE; angiotensin-converting-enzyme. DOAC; direct oral anticoagulant. TIA; transitory ischaemic attack.

### 3.1 Health-related quality of life

The results showed no differences between the female group (FG) and the male group (MG) at baseline or at the end of the study in HRQoL. However, within the FG, improvements were observed in subscales VT (*p* = 0.011), SF (*p* = 0.022), MH *p* = 0.007) and the domain MCS (*p* = 0.022). Also, there were improvements within the MG in subscales BP (*p* = 0.005), GH (*p* = 0.003), VT (*p* = 0.026), SF (*p* = 0.005), RE (*p* = 0.011) and the domain MCS (*p* = 0.018). No improvement was seen in PCS in either of the groups, Table 2.

**Table 2:** Changes in SF-36 over time.

Type of score	FG *n* = 36 *Baseline*	FG *n* = 36 *End of study*	MG *n* = 28 *Baseline*	MG *n* = 28 *End of study*	With-in FG End of study *p-value*	With-in MG End of study *p-value*	Between groups End of study *p-value*
PF	79.4 ± 20.1	80.4 ± 14.4	79.6 ± 17.8	86.8 ± 16.6	0.763	0.289	0.211
RP	59.0 ± 41.8	63.9 ± 41.6	54.5 ± 42.5	69.6 ± 34.9	0.608	0.065	0.437
BP	73.8 ± 25.9	78.0 ± 23.7	62.7 ± 27.9	80.9 ± 23.0	0.379	0.005*	0.066
GH	60.7 ± 22.1	67.3 ± 17.9	54.7 ± 18.9	68.8 ± 20.9	0.094	0.003*	0.198
VT	50.9 ± 19.3	60.8 ± 15.9	52.3 ± 14.9	63.6 ± 18.0	0.011*	0.026*	0.816
SF	69.8 ± 28.9	82.6 ± 19.9	68.3 ± 25.8	85.3 ± 21.3	0.022*	0.005*	0.599
RE	60.2 ± 44.9	75.9 ± 38.7	54.8 ± 43.7	78.6 ± 31.7	0.091	0.011*	0.532
MH	64.8 ± 15.6	72.4 ± 10.2	60.7 ± 15.1	67.1 ± 15.3	0.007*	0.113	0.788
PCS	46.8 ± 9.6	48.0 ± 9.7	45.2 ± 9.1	46.1 ± 9.2	0.221	0.289	0.786
MCS	39.7 ± 11.0	42.6 ± 11.0	38.4 ± 10.1	41.4 ± 10.3	0.022*	0.018*	0.967

Values are mean and standard deviation. * Statistically significant with a *p*-value < 0.05. FG; women group, MG; men group. Dimensions of Short-form health survey (SF-36); PF; physical functioning, RP; role-physical, BP; bodily pain, GH; general health, VT; vitality, SF; social functioning, RE; role-emotional, MH; mental health. Domains of SF-36; PCS; Physical Components Summary, MCS; Mental Components Summary.

### 3.2 Hemodynamic assessments

Between the groups, there were no differences in SBP, DBP or HR at baseline or end of study. Within the FG, there were improvements in SBP (*p* = 0.017) and DBP (*p* < 0.001), but no difference was seen in HR (*p* = 0.285) at the end of study. There was also improvement within the MG in DBP (*p* = 0.026) at end of study, but no differences were seen in SBP (*p* = 0.127) or HR (*p* = 0.760), Table 3.

**Table 3:** Changes in systolic, diastolic blood pressure and heart rate over time.

	FG *n* = 36	FG *n* = 36	MG *n* = 28	MG *n* = 28	With-in FG End of study	With-in MG End of study	Between groups End of study
	Baseline	End of study	Baseline	End of study	*p*-value	*p*-value	*p*-value
SBP/mmHg	137 ± 19	129 ± 18	130 ± 14	127 ± 12	0.017*	0.127	0.314
DBP/mmHg	82 ± 10	74 ± 11	79 ± 9	74 ± 9	<0.001*	0.026*	0.167
HR/minute	63 ± 11	60 ± 12	63 ± 19	64 ± 17	0.285	0.760	0.364

Values are mean and SD. * Statistically significant with a *p*-value < 0.05. FG; women group, MG; men group, mmHg; millimetre of mercury, SBP; systolic blood pressure, DBP; diastolic blood pressure, HR; heart rate.

## 4 Discussion

To our knowledge, this is the first study evaluating if both women and men, with PAF, may improve HRQoL, blood pressure and heart rate after performing MY. Our main findings are that MY improves HRQoL and diastolic blood pressure in PAF, irrespective of gender.

### 4.1 Health-related quality of life

Previous studies describe women, with AF, reporting lower HRQoL than men [5], [21], which is not in line with our results, whereas there were no differences between the groups at baseline or at end of study. However, previous studies have involved all three subgroups of AF and not only PAF as in our study, which could be an explanation of the different result. Also, the literature describes women with AF as older than in our study, and this may perhaps explain the lower HRQoL.

In this study, the men improved their HRQoL in more subscales (BP, GH, VT, SF, RE, MCS) than the women (VT, SF, MH, MCS). The subscales BP and GH reflect the physical function in the questionnaire SF-36, which indicates that MY practices improve it more in men than women but has no effect on PCS.

None of the participants in the studies reported previous experiences of performing yoga. In addition, use of other complementary methods were not analysed in this study. However, the literature describes that women use more complementary methods than men [22] but, in the present study, we cannot conclude that men had used complementary methods less than women.

### 4.2 Blood pressure

SBP and DBP decreased in the FG as well as DBP in the MG, which may support the view that yoga practice (movement, breathing technique and meditation) contributes to an increased balance in the parasympathetic nervous system [8]. The FG group members were older and were diagnosed with hypertension, although not significantly more than the MG. This is in line with other studies [5], [21]. The SBP value was in the reference interval in both groups and there were no differences in the MG. However, the MG had lower SBP than the FG which may reflect that the FG had more hypertension and, furthermore, explain why men are more disposed than women to use medication [5]. Increased blood pressure has an important impact in increasing both recurrent AF [23] and cardiovascular events [24]. Whereas yoga decreased blood pressure, in this study, it may have a role as a prevention strategy in both women and men with PAF.

### 4.3 Heart rate

However, there were no improvements in HR between or within the MG and the FG. The effects of decreased HR have been shown in other studies with yoga and PAF [9] as well as other studies with yoga [25], [26], but not specifically in terms of gender. The result from this study may reflect that the patients in this study were well stabilized regarding medication.

### 4.4 Yoga as a complementary treatment

As mentioned, ESC guidelines emphasize that a comprehensive approach should be considered as a complement to treatment to help reduce modifiable risk factors for the recurrence of AF as well as to increase HRQoL [1]. Patients with AF have suggested yoga as a complementary method to manage symptoms, such as anxiety and worry [27], which may further increase HRQoL [28]. Though yoga is mostly used by women [29], and the results of this study shows that both men and women are in favour of performing MY, this study may encourage men, with PAF, to use complementary methods, such as yoga, to reduce blood pressure and increase HRQoL. Also, as the literature suggests, yoga may have potential effects warranting its implementation in secondary prevention for patients with AF [14]. This study supports the view that both men and women with PAF may experience positive effects of yoga.

Though our study reports improvement in MCS and blood pressure, from MY, in both women and men, we suggest the importance of including stratification by gender in future research.

## 5 Limitations

As HRQoL affects patients with PAF, most prominently in their social lives, the literature strongly recommends measuring HRQoL in studies to evaluate the effects of interventions [1]. However, HRQoL may be difficult to measure given the various factors which may impact it. Factors that reflect HRQoL may occur at different times, which can affect the measurements [30]. In this study, the measurements of HRQoL were equal for both studies and groups and should therefore have no influence on the results.

When measuring HRQoL, the literature recommends including a disease specific HRQoL questionnaire to achieve a comprehensive state of overall HRQoL (i.e., specific symptoms). In this study, only a general HRQoL questionnaire was analysed. Therefore, the analysis of HRQoL could be improved but, despite this, can give guidance and directions regarding differences among patients with PAF after performing yoga. A suggestion for forthcoming studies is to include a disease-specific questionnaire to achieve a deeper analysis of HRQoL in gender differences, among patients with PAF, after performing MediYoga.

## 6 Conclusion

MediYoga appears to improve mental health as well as diastolic blood pressure in both men and women. Thus, an improved mental health may help the person to achieve an improved physical health and therefore an overall improved balance in life. This study suggests, and encourages, the use of complementary methods such as yoga for both men and women.

## Declaration of Competing Interest

The authors declare that they have no known competing financial interests or personal relationships that could have appeared to influence the work reported in this paper.

## Data Availability

Data available on request.
